# Identification of marker genes and cell subtypes in castration-resistant prostate cancer cells

**DOI:** 10.7150/jca.49409

**Published:** 2021-01-01

**Authors:** Xiao-Dan Lin, Ning Lin, Ting-Ting Lin, Yu-Peng Wu, Peng Huang, Zhi-Bin Ke, Yun-Zhi Lin, Shao-Hao Chen, Qing-Shui Zheng, Yong Wei, Xue-Yi Xue, Rong-Jin Lin, Ning Xu

**Affiliations:** 1 Department of Urology, the First Affiliated Hospital of Fujian Medical University, Fuzhou 350005, China.; 2 Department of Nursing, the First Affiliated Hospital of Fujian Medical University, Fuzhou 350005, China.

**Keywords:** single-cell RNA-seq, castration-resistant prostate cancer cells, marker genes, cell subtypes

## Abstract

The diverse tumor cell populations may be the critical roles in relapse and resistance to treatment in prostate cancer patients. This study aimed to identify new marker genes and cell subtypes among castration-resistant prostate cancer (CRPC) cells. We downloaded single-cell RNA seq profiles (GSE67980) from the Gene Expression Omnibus (GEO) database. Principal component (PC) analysis and t-Distributed Stochastic Neighbor Embedding (TSNE) analysis were performed to identify marker genes. CRPC cells were clustered and annotated. GO and Kyoto Encyclopedia of Genes and Genomes (KEGG) analyses among marker genes were performed. A total of 1500 genes with larger standardized variance were obtained. The top 20 genes were demonstrated in each identified 20 PCs. PC with P-value < 0.05 was selected, including PC1, PC7, PC8, and PC14. The TSNE analysis classified cells as two clusters. The top 6 genes in cluster 0 included HBB, CCL5, SLITRK4, GZMB, BBIP1, and PF4V1. Plus, the top 6 genes in cluster 1 included MLEC, CCT8, CCT3, EPCAM, TMPRSS2, EIF4G2. The GO analysis revealed that these marker genes were mainly enriched in RNA catabolic process, translational initiation, mitochondrial inner membrane, cytosolic part, ribosome, cell adhesion molecule binding, cadherin binding, and structural constituent of ribosome. The KEGG analysis showed that these marker genes mainly enriched in metabolism associated pathways, including carbon metabolism, cysteine and methionine metabolism, propanoate metabolism, pyruvate metabolism, and citrate cycle pathways. To conclude, our results provide essential insights into the spectrum of cellular heterogeneity within human CRPC cells. These marker genes, GO terms and pathways may be critical in the development and progression of human CRPC.

## Introduction

Prostate cancer remains the second leading cause of death in men in the US 1-3. Jeremy et al. 4 has conducted a survey concerning global incidence of prostate cancer in developing and developed countries and revealed that the global incidence of prostate cancer has been increasing in most countries, especially in Asia, Northern and Western Europe 5. Although the therapeutic strategies for prostate cancer are varied, the genetic heterogeneity among different prostate cancer patients make the precision medicine gradually important and popular in the treatment of prostate cancer 6-8.

Identification of crucial characterization of intratumoral heterogeneity has gradually become a hot topic recently, for it is crucial for precision cancer management 9, 10. The standard therapy for metastatic prostate cancer remains the androgen deprivation therapy (ADT). However, metastatic prostate cancer patients will inevitably progress to the castration-resistant stage after one or two years' ADT 11. Moreover, the time to the castration-resistant stage from the beginning of receiving ADT in metastatic prostate cancer patients remains diverse among different patients 12. The diverse tumor cell populations may be the critical roles in relapse and resistance to treatment in prostate cancer patients 13. Hence, there is an unmet need for the identification of new biomarkers for guiding the clinical practice.

Previous studies 13-15 have conducted the heterogeneity and molecular complexity among prostate cancer patients. Nevertheless, the exploration of intra-prostatic genomic complexity, especially castration-resistant prostate cancer by utilizing single-cell DNA analysis, has not been reported. Single-cell RNA-Seq enables urologists to identify the difference among each cell 15. Single-cell RNA-Seq can easily illustrate the expression of diverse cellular populations. On the one hand, identification of marker genes through transcriptional data among individual cell is an excellent way to facilitate the understanding of tumor heterogeneity and clinical practice 16, 17. On the other hand, the identification of cell types from a number of heterogeneous cells is an intuitive way of understanding the origin of tumor development.

In this study, we utilized the single-cell RNA seq data downloaded from the Gene Expression Omnibus (GEO) database to illustrate the marker genes and cell subtypes in Castration-resistant prostate cancer cells.

## Methods

### Data download, quality control, data filtering, and normalizing

We downloaded single-cell RNA seq profiles (GSE67980) from the Gene Expression Omnibus (GEO) database. The data utilized in this study were derived from metastatic CRPC patients. The specific inclusion criteria could be reviewed in the orginal article 18. In brief, 18 patients with metastatic prostate cancer were selected as candidates. Only cells with sufficient quality for amplification and next-generation RNA sequencing were included in the following analyses. Finally, data from 15 patients were utilized in this study. We used the package, Seurat, for data analysis. Several adjustments were utilized to increase the sensitivity to outliers specifically. The number of genes in each cell and the number of gene sequencing were evaluated. Quality control and data filtering were performed accordingly. Duplicated gene expression was averaged. Genes expression identified in less than 3 cells were excluded. Cells with less than 50 identified gene sequencing were also excluded. The percentage of the mitochondrial gene was calculated in this study. The number of genes and sequencing, the percentage of the mitochondrial gene were showed by utilizing a violin plot. Cells with less than 50 genes were excluded. Data were then normalized after filtering by the above adjustments. The top 1500 genes with larger standardized variance were selected for the following analyses. The top 10 gene symbols were labeled.

### Principal component (PCA) analysis

PCA was performed by utilizing 1500 screened genes with larger standardized variance. The top 20 principal components were selected for the following analysis. The top 20 genes in each principal component will be plotted. The overview of PCA and heatmaps will be plotted in figures. The P-value of each principal component was generated for the following analyses.

### TSNE analysis and identification of Marker genes

t-Distributed Stochastic Neighbor Embedding (TSNE) analysis was performed after PCA. Cells were classified according to different clusters. The marker genes in different clusters were then demonstrated by utilizing heatmap. The expression level of marker genes in each cluster was demonstrated by utilizing a violin plot. A bubble plot was used to demonstrate the expression level in each cluster.

### Clustering of castration-resistant prostate cancer cells

The castration-resistant prostate cancer cells were clustered using the algorithm developed in R package “Monocle”. The number of clusters was automatically chosen by R package “Monocle”, according to the previously described method 19. Two clusters were identified as follows: cluster 0 and cluster 1.

### Annotation of cell types

We utilized the package of “SingleR” to perform cell annotation. Identified clusters were annotated. Each cell was also annotated.

### GO and KEGG analysis

GO and Kyoto Encyclopedia of Genes and Genomes (KEGG) analyses were conducted by the R package of “digest" and “GOplot”. The names of marker genes were transferred into gene ID. The barplot, bubble plot, circ plot, and cluster plot were used to demonstrate the results in different directions.

## Results

### Data download, quality control, data filtering, and normalizing

**Figure 1:**
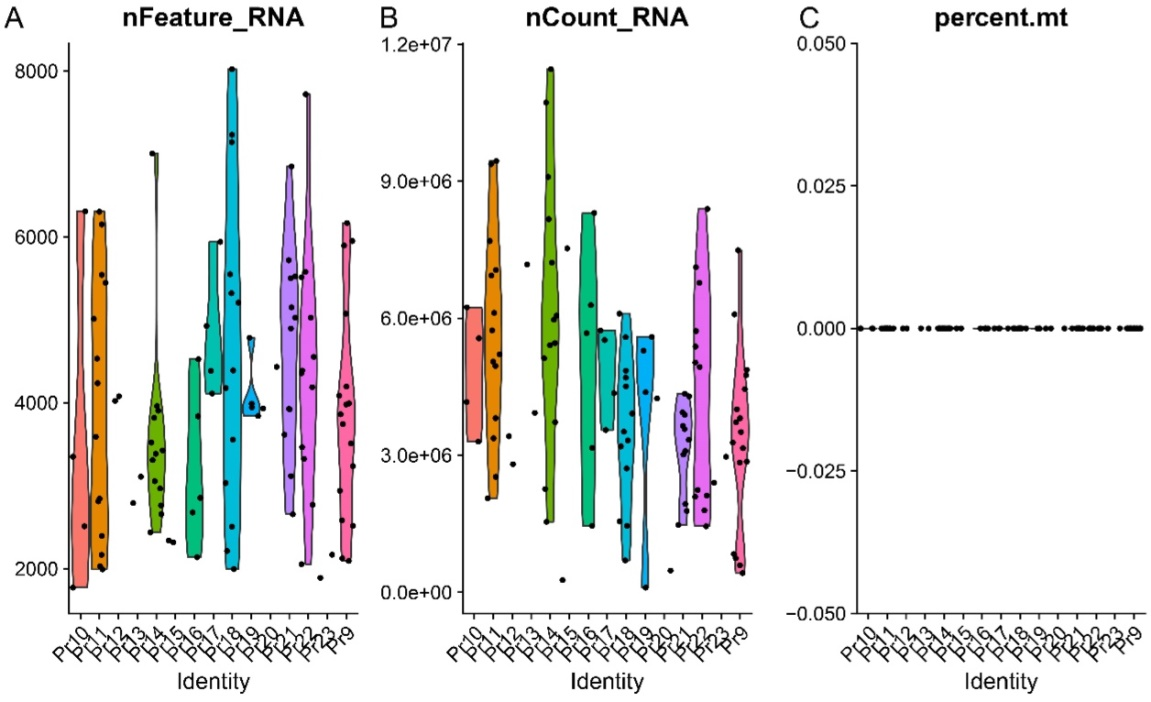
The outcomes of quality control. (A) The distribution of identified genes in each cell. (B) The distribution of identified gene sequencing. (C) The percentage of mitochondrial gene. The X-axis from left to right represents each screen donors; the dots in the Figures represent each evaluated cell. The Y-axis reflects the number of genes in each cell.

A total of fifteen donors contains 108 single cells were identified. The X-axis from left to right represents each screen donors; the dots in the Figures represent each evaluated cell. The Y-axis reflects the number of genes in each cell. The distribution of genes in each cell was demonstrated in Figure 1A. The cells with genes more than 8000 and less than 2000 were excluded in this study). The distribution of identified gene sequencing was demonstrated in Figure 1B. No mitochondria gene was screened in this study (Figure 1C).

**Figure 2:**
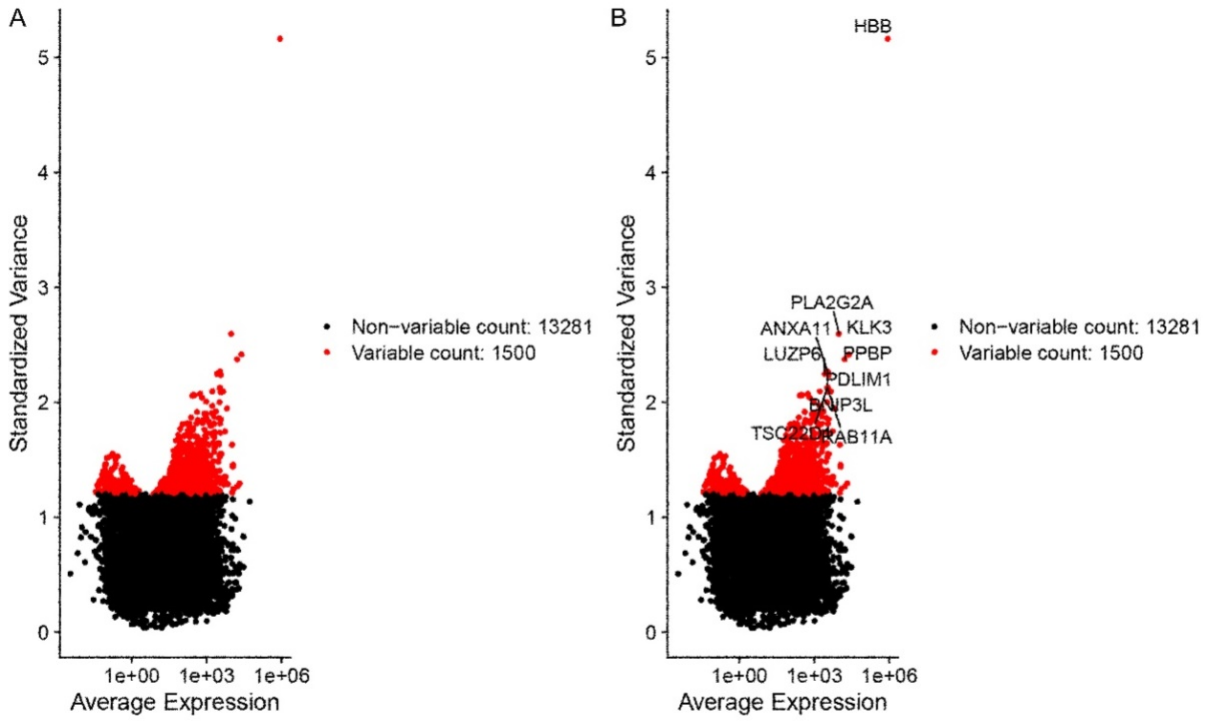
The outcomes of data filtering and normalizing. (A) A total of 1500 genes with larger standardized variance. (B) The top 10 standardized variance genes in 1500 genes were demonstrated. The X-axis represents the average expression of each gene. The Y-axis represents standardized variance.

The X-axis represents the average expression of each gene. The Y-axis represents standardized variance. Genes with larger standardized variance were screened and selected for the following analyses, for these genes represent the heterogeneity among cells. A total of 1500 genes with larger standardized variance were obtained (Figure 2A). The top 10 standardized variance genes were demonstrated in Figure 2B.

### Principal component analysis (PCA)

**Figure 3:**
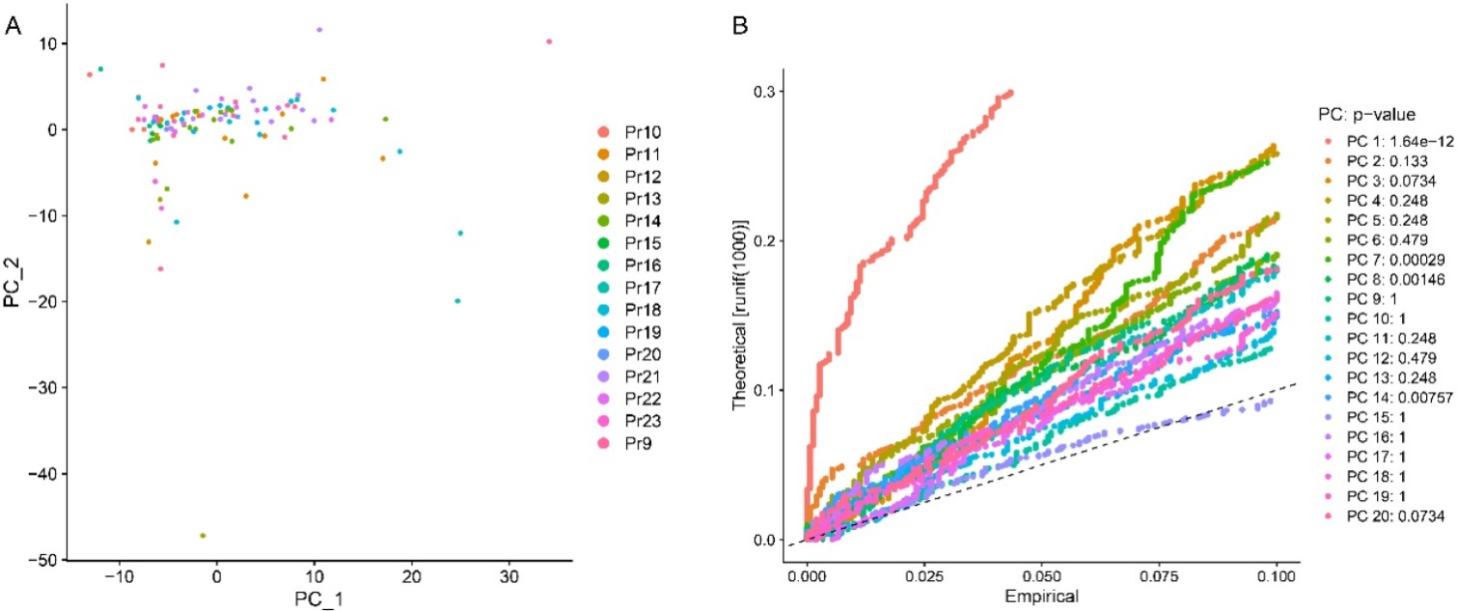
The results of principal component (PC) analysis. (A) The overview distribution of cells of each donor in PC1 and PC2. (B) The P value of each PC.

**Figure 4:**
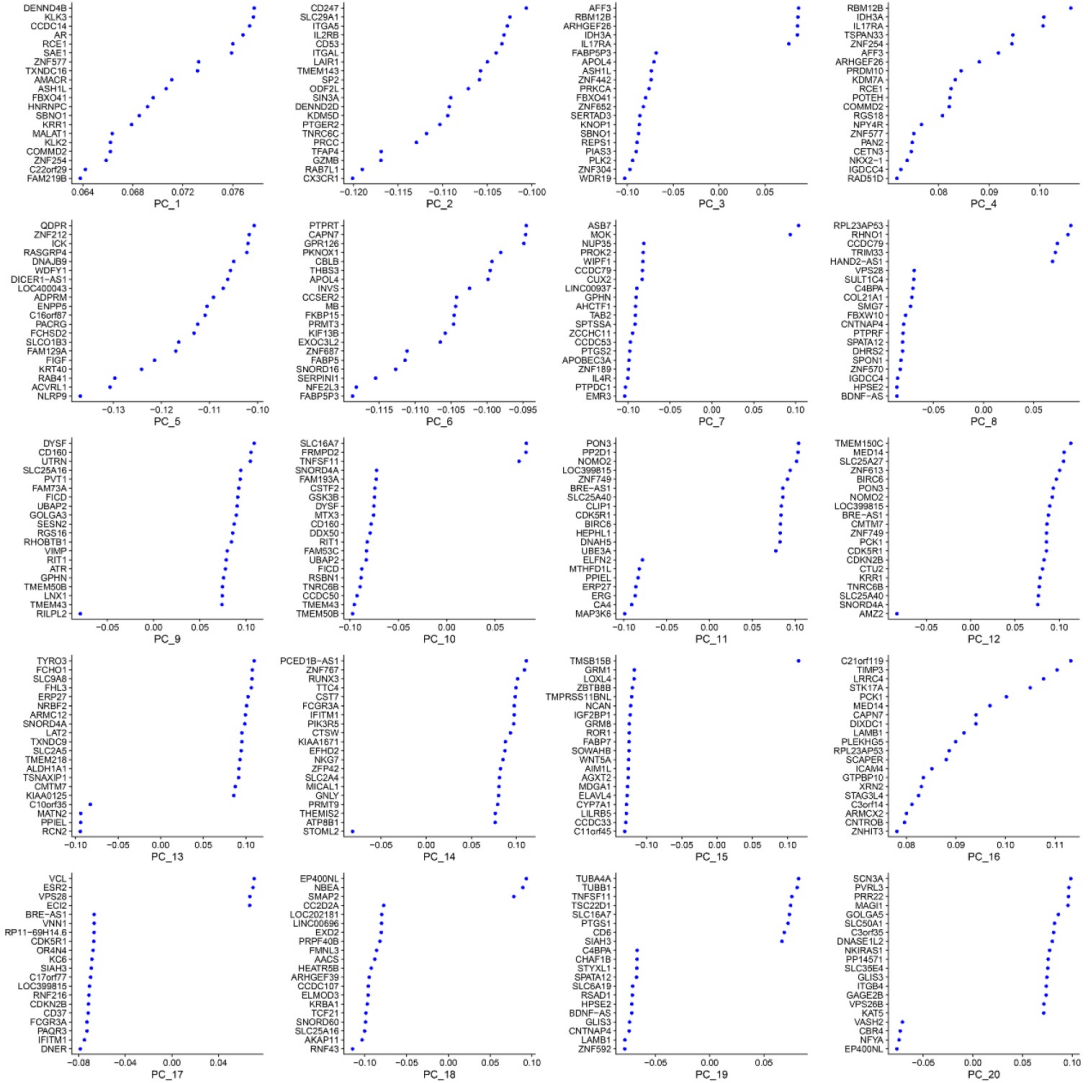
The top 20 principal components (PCs) and top 20 genes expressed in each PC.

**Figure 5:**
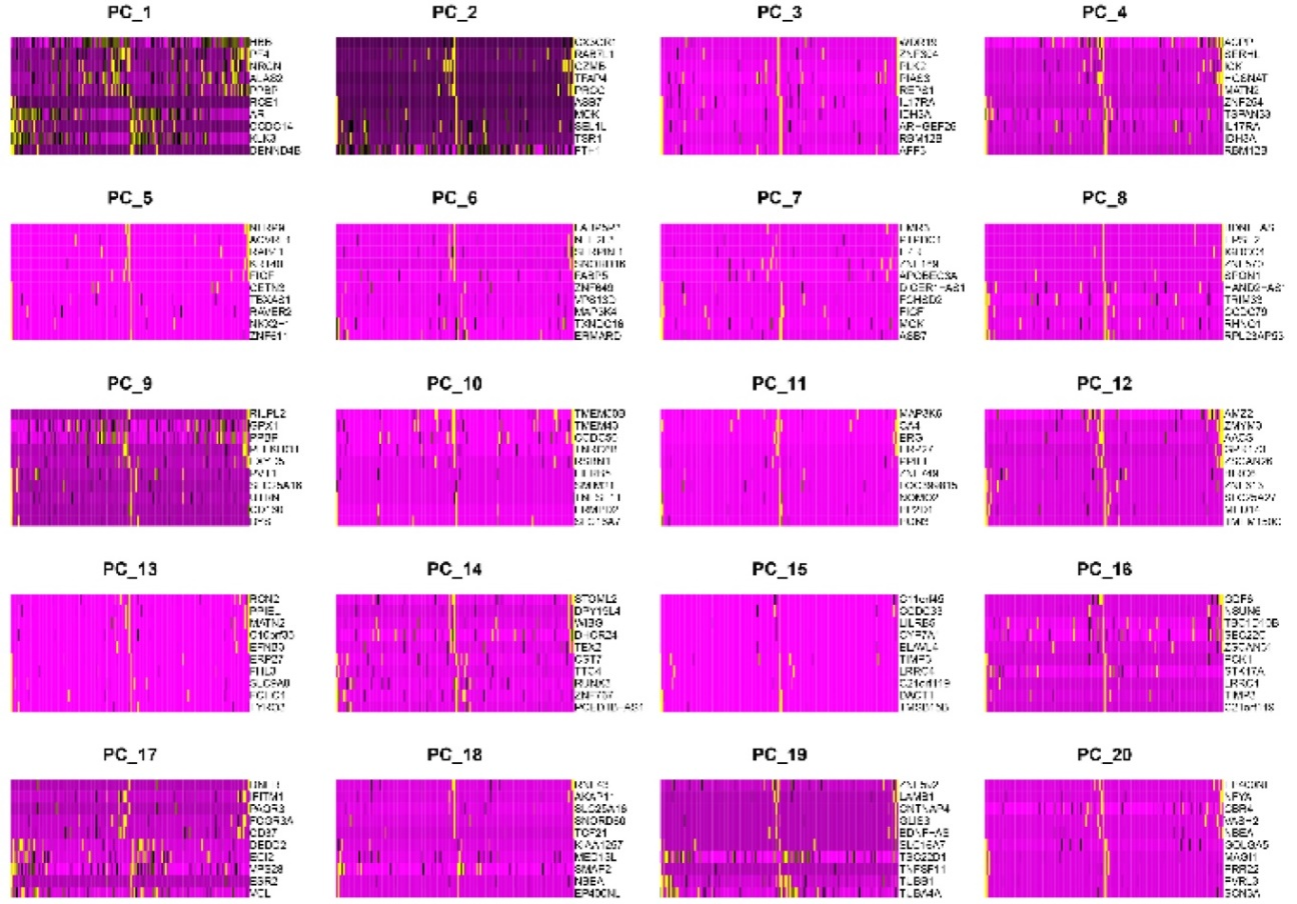
The heatmaps of the top 20 principal components.

The most variable genes were utilized to compute the main sources of variability in this study, as demonstrated in the PCA. The aim of PCA was to identify the features in these most variable genes. PC1 and PC2 were the components with the most predominant variations. The overview distribution of cells of each donor in PC1 and PC2 were demonstrated in Figure 3A and the P-value for each PC was demonstrated in Figure 3B. Each color demonstrated in the Figure 3A was represented to different dornors. Each dot in the Figure 3A was represented to each cell of different dornors. After PCA, a total of 20 PCs were demonstrated in Figure 4. The top 20 genes expressed in each PC were also demonstrated in Figure 4. The PC1 was regarded as one of the most predominant principal components in PCA. The top 20 genes were listed as follow: DENND4B, KLK3, CCDC14, AR, RCE1, SAE1, ZNF577, TXNDC16, AMACR, ASH1L, FBXO41, HNRNPC, SBNO1, KRR1, MALAT1, KLK2, COMMD2, ZNF254, C22orf29, FAM219B. The heatmaps of the top 20 PCs were shown in Figure 5. The heatmap of representative genes of PC1 listed in Figure 5 were HBB, PF4, NRGN, ALAS2, PPBP, RCE1, AR, CCDC14, KLK3, DENND4B. PC with P-value < 0.05 was selected for the following analysis. The PCA results demonstrated that PC1, PC7, PC8, and PC14 were essential PCs.

### Cell type classification by TSNE analysis and identification of marker genes

**Figure 6:**
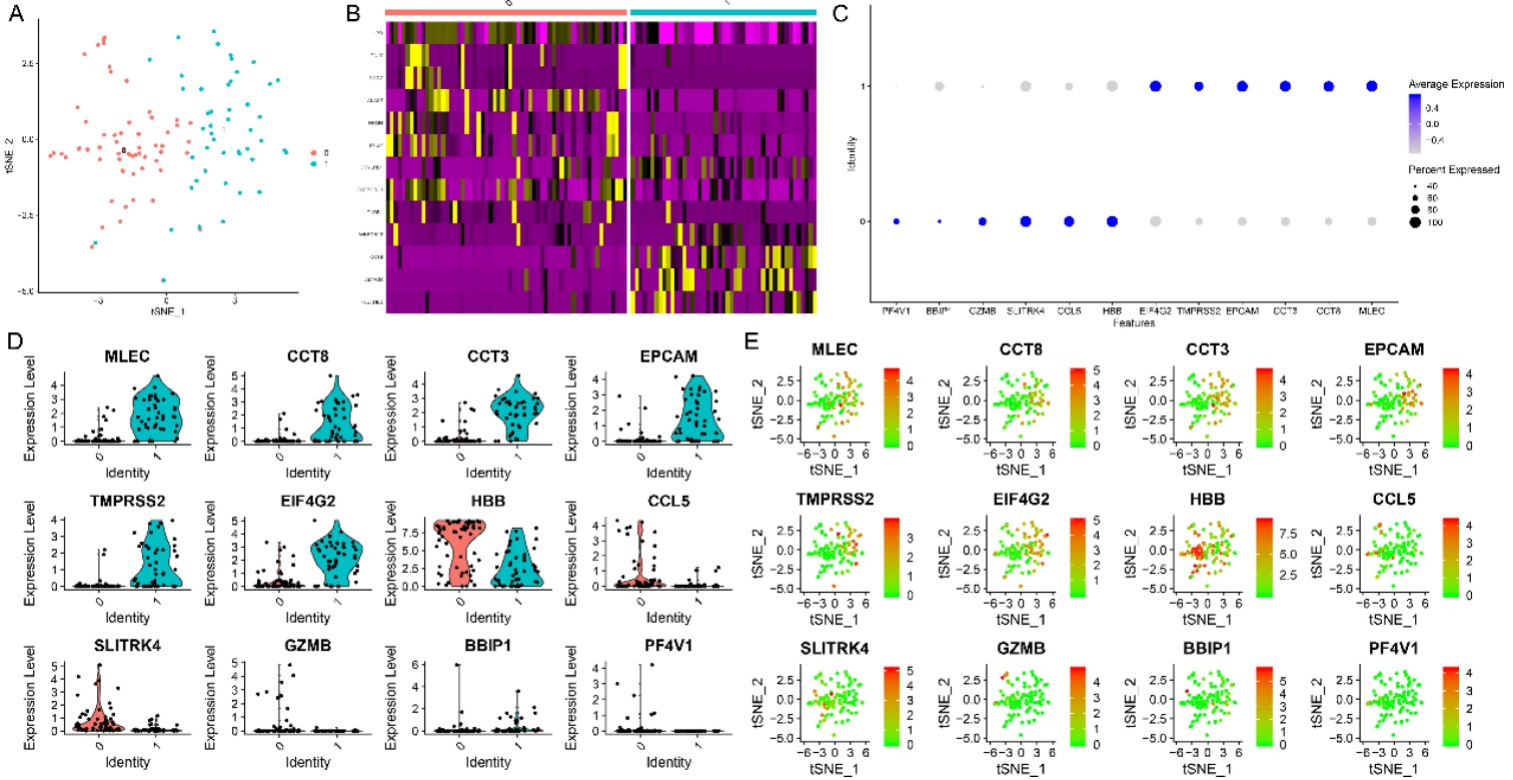
The results of TSNE analysis and marker genes. (A) The results of the TSNE analysis. (B) The heatmaps demonstrated the critical genes in cluster 0 and cluster 1. (C) The top 6 genes with the highest logFC in cluster 0 and cluster 1. (D) The violin plot demonstrated the expression level in each cluster. (E) The scatter plot showed the distribution of each cell in selected marker genes.

The results of the TSNE analysis classified cells as two clusters (Figure 6A). After annotation, we found that these cells could be classified as cluster 0: monocytes, and cluster 1: epithelial cells. Red color represents cluster 0 and green color represents cluster 1. The heatmaps demonstrated the critical genes in cluster 0 and cluster 1 (Figure 6B). The representative genes in heatmaps were listed as follow: HBB, GNLY, NKG7, ALAS2, NRGN, PPBP, DYNLRB1, TUBB1, WBSCR22, CCT8, AMACR, PLEKHB2. Then, the top 6 genes with the highest logFC in cluster 0 were demonstrated in Figure 6C, including HBB, CCL5, SLITRK4, GZMB, BBIP1, and PF4V1. Plus, the top 6 genes with the highest logFC in cluster 1 were demonstrated in Figure 6C, including MLEC, CCT8, CCT3, EPCAM, TMPRSS2, and EIF4G2. For the sake of evaluating the expression level of these marker genes in both cluster 0 and cluster 1, the violin plot (Figure 6D) was utilized to demonstrate the expression level in each cluster. The results of violin plots demonstrated that the expression levels of MLEC, CCT8, CCT3, EPCAM, TMPRSS2, and EIF4G2 were higher in cluster 1 when compared with cluster 0. In contrast, the expression level of CCL5 and SLITRK4 were higher in cluster 0 when compared with cluster 1. The scatter plot (Figure 6E) was used to show the distribution of each cell in selected marker genes.

### GO and KEGG analysis

**Figure 7:**
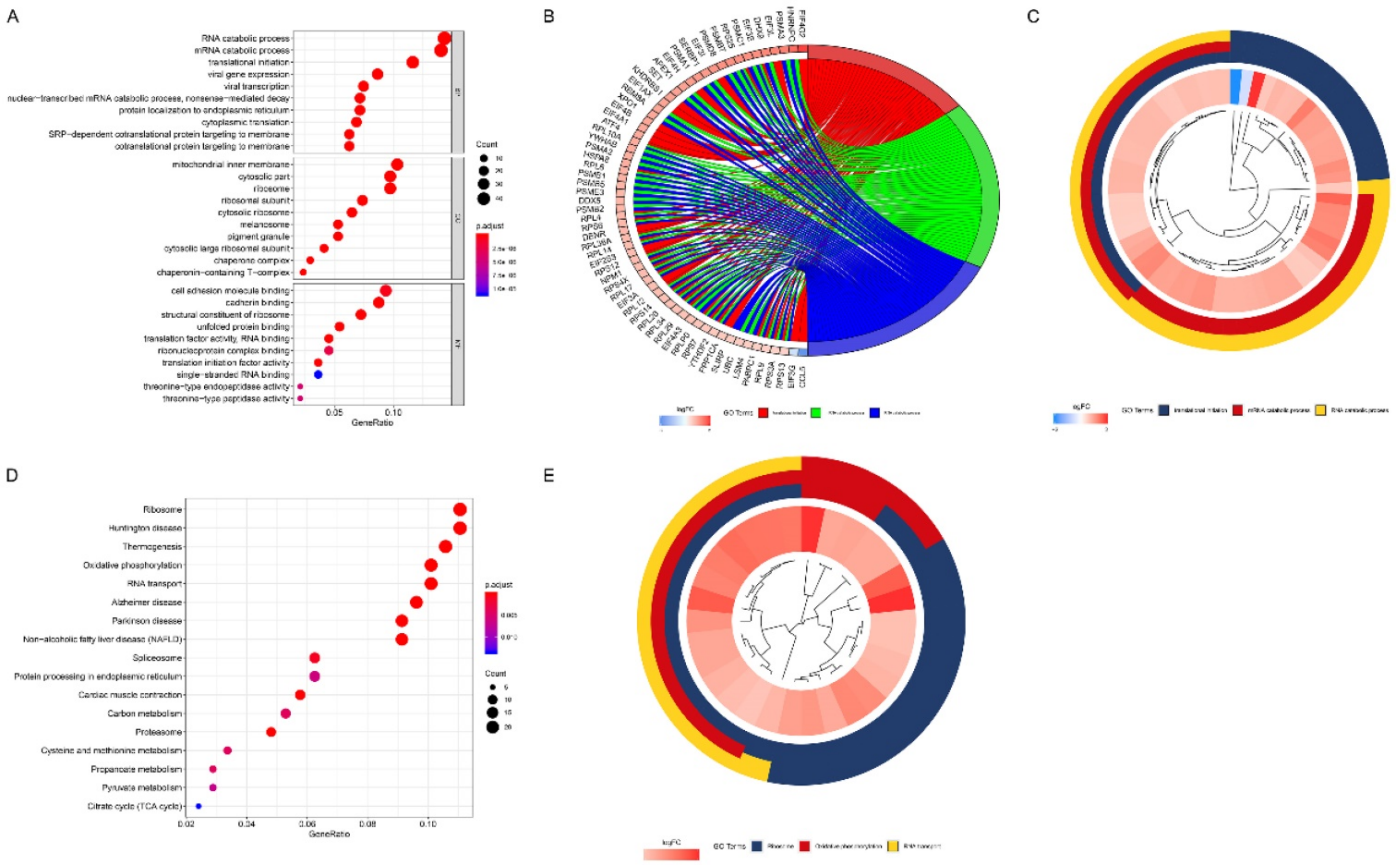
The results of GO and KEGG analysis. (A) The results of GO analysis among identified marker genes. (B) The top three enriched GO terms were demonstrated in the cluster plot. (C) The genes enriched in each top three GO terms were shown in the chord plot. (D) The results of KEGG analysis among identified marker genes. (E) The top three enriched KEGG pathways were demonstrated in the cluster plot.

The results of GO analysis revealed that these marker genes were mainly enriched in RNA catabolic process, translational initiation, mitochondrial inner membrane, cytosolic part, ribosome, cell adhesion molecule binding, cadherin binding, and structural constituent of ribosome (Figure 7A). The top three enriched GO terms were demonstrated in the cluster plot in Figure 7B. The innermost layer represents gene clustering; the interlayer represents the fold change; the outermost layer represents the GO terms. The genes enriched in each top three GO terms were shown in the chord plot in Figure 7C. The left part of Figure 7C represents genes (different color represents different fold change); the right part of Figure 7C represents GO terms; the chord represents the association between genes and GO terms.

The results of KEGG analysis showed that these marker genes mainly enriched in metabolism associated pathways, including carbon metabolism, cysteine and methionine metabolism, propanoate metabolism, pyruvate metabolism, and citrate cycle pathways (Figure 7D). The top three enriched KEGG pathways were demonstrated in the cluster plot (Figure 7E).

## Discussion

Cancers commonly exhibit tumor heterogeneity in the modality of distinguishable phenotypic characteristics, like different cellular morphology, metabolic types, and gene alterations 9. It is essential to classify cell populations into each subtype according to single-cell expression data for analyzing tumor heterogeneity. Our scRNAseq analysis of the human castration-resistant prostate cancer cells to illustrate the marker genes and substantial cell subtypes. Strikingly, this study identified 1500 genes with significantly standardized variance, which reflects that the expression pattern of these 1500 genes was virtually different among each castration-resistant prostate cancer cell. Plus, we identified four essential PC, like PC1, PC7, PC8, and PC14. The TSNE analysis was used to identify marker genes. The cells were classified into two clusters by the TSNE analysis. The top 3 genes with the highest logFC in cluster 0 were HBB, CCL5, and SLITRK4. Plus, the top 3 genes with the highest logFC in cluster 1 were MLEC, CCT8, and CCT3.

Maman et al. 20 reported that HBB could suppress the growth and metastasis. Plus, Onda et al. 21 demonstrated that the expression of HBB in anaplastic thyroid cancer was decreased and recovery of its expression inhibited cell growth. However, the role of HBB in prostate cancer has not been reported yet. CCL5 has been reported extensively in many kinds of cancer by previous studies, like breast cancer 22-24, non-small-cell lung cancer 25, 26, colorectal cancer 27, 28. Nevertheless, the mechanism of CCL5 in prostate cancer has not been studied very well. Ji et al. 29 demonstrated that miR-589-5p regulates prostate cancer cell viability and metastasis by targeting CCL-5. Xiang et al. 30 showed that Infiltrating CD4+ T cells attenuate chemotherapy sensitivity in prostate cancer via CCL5 signaling. Yeh et al. 31 demonstrated that via reducing CCL5 in the tumor microenvironment, estrogen receptor α in cancer-associated fibroblasts could suppress prostate cancer invasion. Unfortunately, the role of SLITRK4 and MLEC in cancer has not been illustrated yet. Qiu et al. 32 reported that overexpression of CCT8 might be associated with poor outcome of glioma and could regulate the proliferation and invasion of glioblastomas. Liu et al. 33 showed that CCT3 acts upstream of YAP and TFCP2 as a potential target and tumor biomarker in liver cancer.

After that, we performed GO and KEGG analyses to explore the function of these marker genes. The results demonstrated that these marker genes were mainly enriched in RNA catabolic process, translational initiation, mitochondrial inner membrane, cytosolic part, ribosome, cell adhesion molecule binding, cadherin binding, and structural constituent of ribosome. Also, the results of KEGG analysis showed that these marker genes mainly enriched in metabolism associated pathways, including carbon metabolism, cysteine and methionine metabolism, propanoate metabolism, pyruvate metabolism, and citrate cycle pathways.

Exploring the earliest phases of the origins of castration-resistant prostate cancer is essential for improving the methods of cancer early detection and preventing cancer progression before it transfers into a life-threatening disease 9, 34-36. Previous studies 19, 37 have illustrated that intra-tumor heterogeneity is central to the biological behaviors of tumors. For instance, nearly all patients with metastatic prostate cancer patients will evitably progress to the castration-resistant stage after one or two years' endocrine therapy 11. Also, the time to castration-resistant from the start of endocrine therapy remains diverse among different patients. Tumors are comprised of variable proportions of malignant, stromal, and immune components. According to the results of cluster trajectory, cluster 0 was annotated as NK cells, and cluster 1 was annotated as epithelial cells.

To conclude, our results provide essential insights into the spectrum of cellular heterogeneity within the human castration-resistant prostate cancer cells. The identification of marker genes, GO terms, and KEGG pathways may play critical roles in the development and progression of human castration-resistant prostate cancer.
